# Supplementary figures and images for: Banp regulates DNA damage response and chromosome segregation during the cell cycle in zebrafish retina
Source: eLife. 2022 Aug 9;11:e74611. doi: 10.7554/eLife.74611 (PMC9363121; doi:10.7554/eLife.74611)

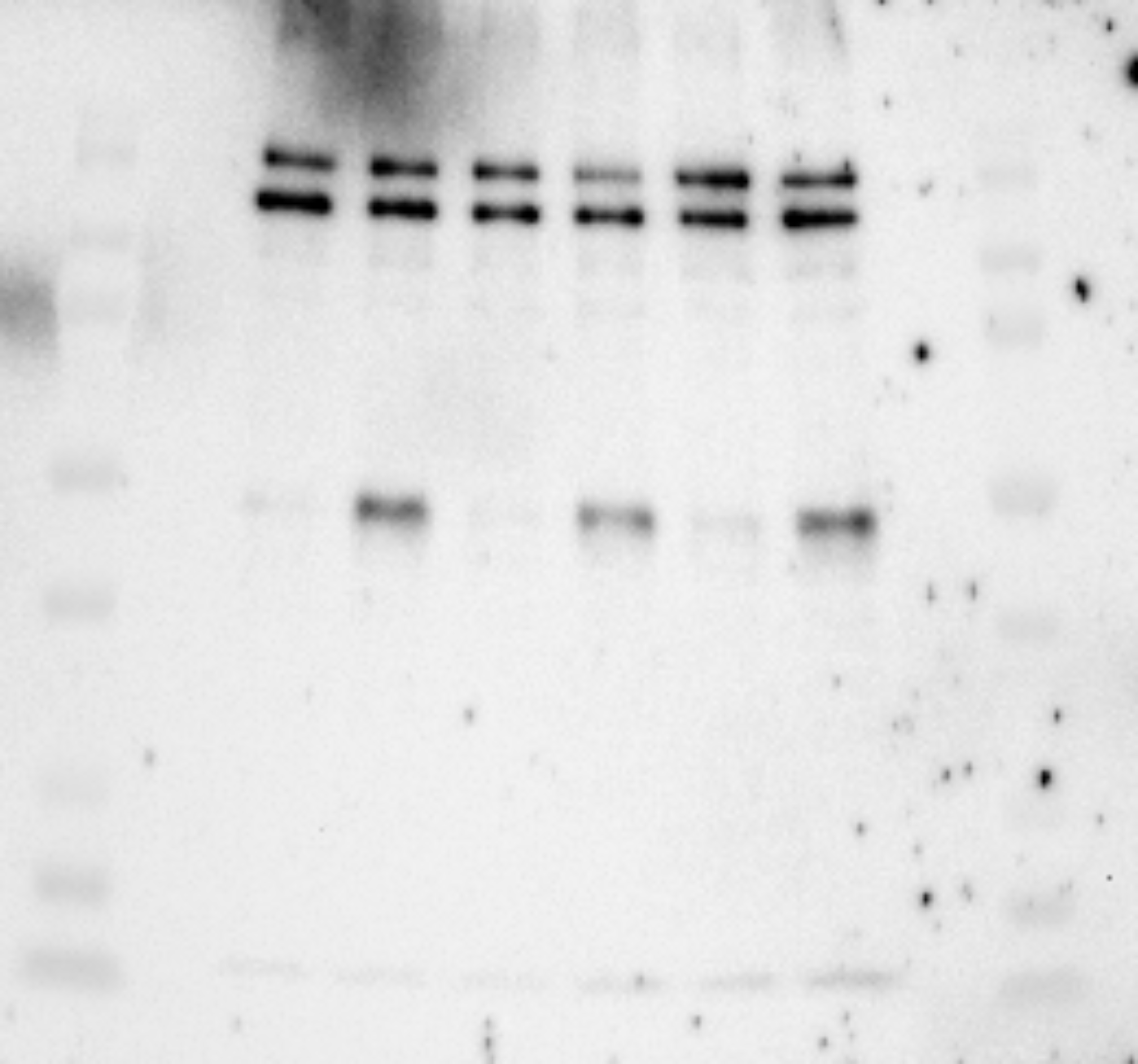

Supplement: Figure 3—source data 2. [file elife-74611-fig3-data2.zip › Data for Figure 3D (tp53_original blot).tif]

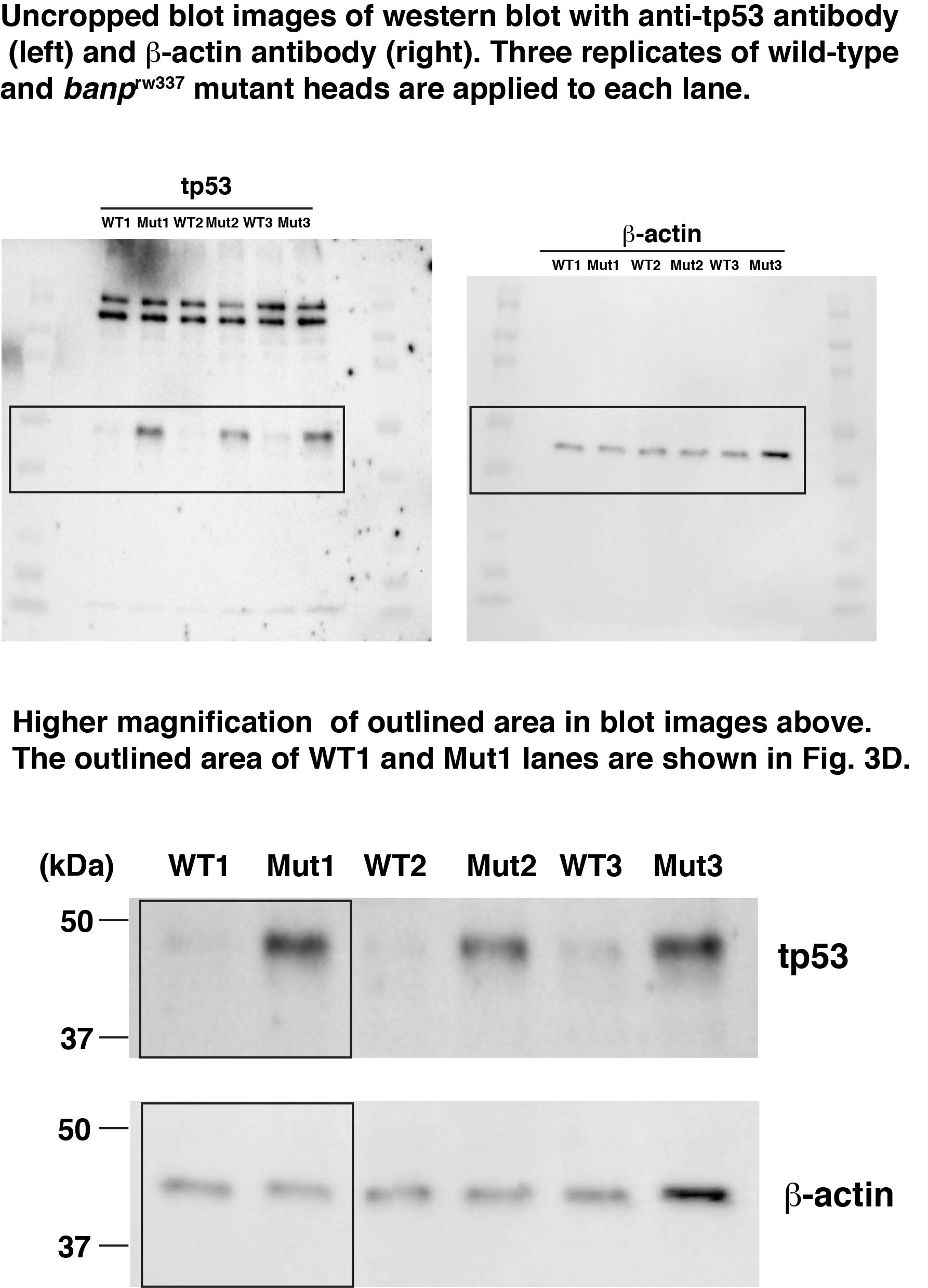

Supplement: Figure 3—source data 2. [file elife-74611-fig3-data2.zip › Data for Figure 3D (uncropped blot image).jpg]

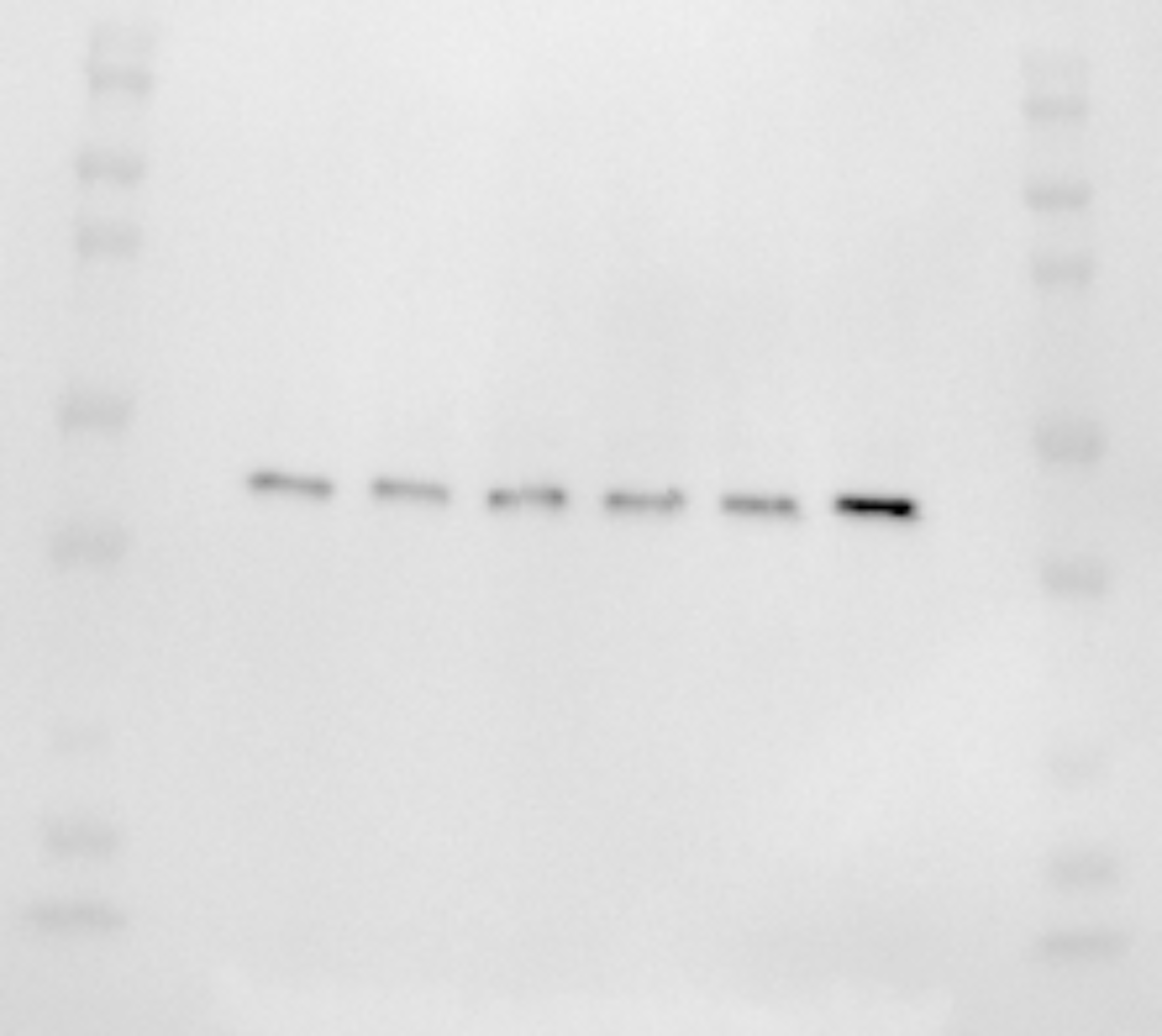

Supplement: Figure 3—source data 2. [file elife-74611-fig3-data2.zip › Data for Figure 3D (beta-actin_oroginal blot).tif]
