# Supplementary material for: Banp regulates DNA damage response and chromosome segregation during the cell cycle in zebrafish retina
Source: eLife. 2022 Aug 9;11:e74611. doi: 10.7554/eLife.74611 (PMC9363121; doi:10.7554/eLife.74611)
Supplement: Transparent reporting form [file elife-74611-transrepform1.docx]

***eLife’s* transparent reporting form**

We encourage authors to provide detailed information *within their submission* to facilitate the interpretation and replication of experiments. Authors can upload supporting documentation to indicate the use of appropriate reporting guidelines for health-related research (see [EQUATOR Network](http://www.equator-network.org/%20)), life science research (see the [BioSharing Information Resource](https://biosharing.org/)), or the [ARRIVE guidelines](http://www.plosbiology.org/article/info:doi/10.1371/journal.pbio.1000412) for reporting work involving animal research. Where applicable, authors should refer to any relevant reporting standards documents in this form.

If you have any questions, please consult our Journal Policies and/or contact us: [editorial@elifesciences.org](mailto:editorial@elifesciences.org).

**Sample-size estimation**

- You should state whether an appropriate sample size was computed when the study was being designed
- You should state the statistical method of sample size computation and any required assumptions
- If no explicit power analysis was used, you should describe how you decided what sample (replicate) size (number) to use

When the study was designed, sample size was not calculated, and no explicit power analysis was utilized. The sample size was determined based on standards in the field. All the experimental data are compared between zebrafish siblings and mutants. A minimum of 3 biological replicates were used in all the experiments performed. The number of biological replicates is represented in figure legends.

Please outline where this information can be found within the submission (e.g., sections or figure legends), or explain why this information doesn’t apply to your submission:

**Replicates**

- You should report how often each experiment was performed
- You should include a definition of biological versus technical replication
- The data obtained should be provided and sufficient information should be provided to indicate the number of independent biological and/or technical replicates
- If you encountered any outliers, you should describe how these were handled
- Criteria for exclusion/inclusion of data should be clearly stated
- High-throughput sequence data should be uploaded before submission, with a private link for reviewers provided (these are available from both GEO and ArrayExpress)

Please outline where this information can be found within the submission (e.g., sections or figure legends), or explain why this information doesn’t apply to your submission:

Each experiment was performed multiple times until we obtained at least three biological replicates. For histology, an individual embryo is considered as one biological replicate. Each experiment consists of a minimum of 3 or more biological replicates. The number of biological replicates is shown in figure legends. The data variation is represented in the graphs with two-sided standard deviation. For ATAC-seq, RNA-seq, western blotting and qRT-PCR, a pool of embryo heads is considered as one biological replicate. The number of embryonic heads was used n=3 for ATAC-seq, n=7 for RNA=seq, n=7 for western blotting and n=7 for qRT-PCR. Each experiment consists of 3 biological replicates. Raw RNA-seq and ATAC-seq datasets of *banp^rw337^* mutant and siblings are available at DDBJ Sequence Read Archive (DRA012572).

**Statistical reporting**

- Statistical analysis methods should be described and justified
- Raw data should be presented in figures whenever informative to do so (typically when N per group is less than 10)
- For each experiment, you should identify the statistical tests used, exact values of N, definitions of center, methods of multiple test correction, and dispersion and precision measures (e.g., mean, median, SD, SEM, confidence intervals; and, for the major substantive results, a measure of effect size (e.g., Pearson's r, Cohen's d)
- Report exact p-values wherever possible alongside the summary statistics and 95% confidence intervals. These should be reported for all key questions and not only when the p-value is less than 0.05.

Please outline where this information can be found within the submission (e.g., sections or figure legends), or explain why this information doesn’t apply to your submission:

Significance of differences between groups (wild-type sibling vs mutant) were calculated using no-parametric t-tests. Mann Whitney (two tailed) test was used for stringency, if the data is not normally distributed. Unpaired t-test (two tailed) was used if the data is distributed normally. Two-way ANOVA was used for multiple comparisons. All data are presented as means ± SDs. A p-value of 0.05 was considered statistically significant. The raw data and exact p-value is available in the source DATA file.

(For large datasets, or papers with a very large number of statistical tests, you may upload a single table file with tests, Ns, etc., with reference to sections in the manuscript.)

**Group allocation**

- Indicate how samples were allocated into experimental groups (in the case of clinical studies, please specify allocation to treatment method); if randomization was used, please also state if restricted randomization was applied
- Indicate if masking was used during group allocation, data collection and/or data analysis

Please outline where this information can be found within the submission (e.g., sections or figure legends), or explain why this information doesn’t apply to your submission:

We do not have group allocation in our study.

**Additional data files (“source data”)**

- We encourage you to upload relevant additional data files, such as numerical data that are represented as a graph in a figure, or as a summary table
- Where provided, these should be in the most useful format, and they can be uploaded as “Source data” files linked to a main figure or table
- Include model definition files including the full list of parameters used
- Include code used for data analysis (e.g., R, MatLab)
- Avoid stating that data files are “available upon request”

We provide source data for histogram of Figs. 2C, 2D, 3C, 3E, 3F, 3H, 4A, 4D, 4E, 4F, 5A, 5C, 5F, 5H, and Fig. 1-figure supplement 4D; Fig. 1-figure supplement 5B; Fig. 2-figure supplement 1C, 1F, 1G, 1I, 1K; Fig. 4-figure supplement 1B, 1D, 1F, 1H, 1J; Fig. 6-figure supplement 2C; Source data for western blot of Fig. 3D are provided in a Zip format.

Please indicate the figures or tables for which source data files have been provided:
